# Exploring Perception of Vibrations from Rail: An Interview Study

**DOI:** 10.3390/ijerph14111303

**Published:** 2017-10-26

**Authors:** Laura Maclachlan, Kerstin Persson Waye, Eja Pedersen

**Affiliations:** 1 Department of Public Health and Community Medicine, University of Gothenburg, Box 414, 40530 Göteborg, Sweden; kerstin.persson.waye@amm.gu.se; 2 Department of Architecture and the Built Environment, Lund University, Box 118, 22100 Lund, Sweden; eja.pedersen@arkitektur.lth.se

**Keywords:** sustainable transport, public health, quality of life, rail vibrations, annoyance

## Abstract

Rail transport is an environmentally responsible approach and traffic is expected to increase in the coming decades. Little is known about the implications for quality of life of populations living close to railways. This study explores the way in which vibrations from rail are perceived and described by these populations. The study took place in the Västra Götaland and Värmland regions of Sweden. A qualitative study approach was undertaken using semi-structured interviews within a framework of predetermined questions in participants’ homes. A 26.3% response rate was achieved and 17 participants were interviewed. The experience of vibrations was described in tangible terms through different senses. Important emerging themes included habituation to and acceptance of vibrations, worry about property damage, worry about family members and general safety. Participants did not reflect on health effects, however, chronic exposure to vibrations through multimodal senses in individual living environments may reduce the possibility for restoration in the home. Lack of empowerment to reduce exposure to vibrations was important. This may alter individual coping strategies, as taking actions to avoid the stressor is not possible. The adoption of other strategies, such as avoidance, may negatively affect an individual’s ability to cope with the stressor and their health.

## 1. Introduction

The European Commission’s 2011 Transport White Paper indicates that railway forms a core component of the move to achieving more sustainable modes of transport [1]. The aim is to increase railway capacity by 50% [2]; an environmentally responsible approach that will contribute to a reduction in greenhouse gas emissions and therefore benefit public health. Nevertheless, on a more local level this increase in rail traffic may have negative implications for the quality of life and individual health of those populations whose living environment is in close proximity to railways.

The relationship between people and their surrounding environment can be perceived in terms of physiology, psychology and ethnology [3,4]. Resources available in our environment affect our quality of life, not just through their physical presence but also through the subsequent demands they place on us which in turn affects our behaviour [4]. The environment in which we live, our home, has an important role to play in our emotional, physical and mental well-being and sense of security. Viewed as a sanctuary, our home is also a space in which we can seek restoration and respite from daily stresses. If the opportunity to restore and relax is removed due to the presence of external stressors, this can have negative implications for our well-being and health.

Environmental stress comes in response to environmental overload, referred to as stressors, and results in acute physiological changes which can be positively or negatively mediated by an individual’s emotional response. Thought to be secondary to the release of catecholamines triggered by emotional stress these physiological changes lead to upregulation of the autonomic nervous system resulting in an increase in heart rate, heart contractility and blood pressure [5]. This response has been shown following exposure to naturally occurring environmental stressors where the experience of major earthquakes has been associated with increased mortality in individuals with existing coronary heart disease [6]. Higher resting heart rates have also been reported in individuals experiencing financial loss and isolation from family after forced relocation from the home and neighbourhood following an earthquake, which may result from psychological stress [7] from a negative emotional response.

The Cognitive Activation Theory of Stress described by Ursin and Eriksen [8,9] incorporates four aspects of stress: stress stimuli; stress experience; the non-specific, general stress response; and experience of the stress response. Activation of the stress pathway is necessary for survival, as can be seen by the physiological changes in earthquakes, which show a “flight or fight” response. However, chronic stress can lead to an imbalance in homeostasis and dysfunction of the hypothalamic-pituitary-adrenal (HPA) axis. Allostasis is the adaptive processes that act to achieve equilibrium through psychological or behavioural changes [10]. Allostatic overload is the cumulative result of allostasis and is associated with raised cortisol, a stress hormone [11]. A sustained stress response leads to increased arousal and wakefulness and is associated with elevated cortisol levels.

Longer-term exposure to noise causes annoyance and disrupted sleep and may result in physiological changes, including an increased prevalence of cardiovascular disease, such as hypertension, myocardial infarction and stroke [12]. At a global level, the burden of disease from environmental noise is significant and it is suggested that in European countries 1.8% of myocardial infarctions can be attributed to exposure to road traffic noise [13]. These changes may be mediated by both a direct pathway, where physiological changes are seen with exposure, and an indirect pathway, secondary to an emotional response or annoyance. The concept of annoyance is broad. In the context of the surrounding environment it can be associated with irritation, concern, discomfort and uneasiness and even loss of control and negative perception of the originating source. Noise annoyance can be defined in terms of different theoretical constructs centred on the view of annoyance as an emotion, a result of disturbance, an attitude, knowledge or a result of rational decisions [14].

It is difficult to evidence the impact that annoyance—“a feeling of displeasure associated with any agent or condition, known or believed by an individual or group to adversely affect them” [15]—may have in disease pathogenesis. However, when considered in the context of the World Health Organization’s (WHO) definition of health as “a state of complete physical, mental and social well-being and not merely the absence of disease or infirmity” [16], annoyance can be viewed as a disruptor of this well-being. The role that annoyance has to play in disrupting the body’s normal physiological response, or homeostasis, may also be significant. Furthermore, annoyance has been associated with both depression and anxiety [17], although causality cannot be determined from cross-sectional studies.

Exposure to vibrations from railways has been associated with higher annoyance levels. Freight trains in particular emit higher levels of noise and vibrations and may act as a warning, eliciting fear and arousal in readiness for a flight-or-fight response. Dose-response relationships between vibration levels and reported annoyance have, however, been difficult to establish. Higher vibration levels were associated with higher frequency of self-reported annoyance in the Netherlands, though not in Poland, in the European Union-funded Cargovibes study [18]. Furthermore, multiple exposure to both noise and vibration has been suggested to increase the likeliness of annoyance in an epidemiologic survey conducted in Pisa, Italy [19]. The influence of other external factors on annoyance to vibration has only been evaluated in a handful of studies [20], though it is likely that some of the moderating factors known from noise studies are relevant also in the case of vibrations, such as the characteristics of the exposure [21,22] or of so-called “non-noise related factors”, such as visibility of the noise source [23,24,25,26]. Factors associated specifically with the way in which vibrations are perceived are demographics, concern of damage to property, and an expectation of increased future vibration [20].

Research into the sequelae of exposure to transport noise has gained momentum over recent decades and these are well studied. Consequently, there is good understanding of annoyance and disturbance to rail noise [27,28]. Research into its counterpart, vibrations from vehicles, has been slower to come to the fore, in part due to technical difficulties in modelling exposure. Little is known about the way in which those populations regularly exposed to vibrations from rail traffic over the long-term describe and perceive them or about the possible implications of this environmental stressor, specifically in terms of an emotional and physiological response. The first stage of a larger project examining the impact of exposure to vibrations—EpiVib—was to explore the way in which people describe vibrations and provide a solid knowledge base in an area in which little is known. For this, a qualitative study design using in-depth interviews with people exposed to vibrations from railway in their daily lives was found suitable.

The aim of the study was to investigate the perception of vibrations from railways by exploring the way in which vibrations, and experiences connected to the vibrations, were described by locals living close to railways.

Two research questions formed the basis for the project. The first referred to pre-set outcomes and investigated the way in which participants talked about vibrations in their home, what happens in the house and how this made them feel. The second research question was aimed at exploring other emerging themes and categories that were not known about at the start of the study.

## 2. Methods

The study design was qualitative—a design that is considered hypothesis generating (in contrast to the hypothesis testing of quantitative) [29]. It was the most appropriate design in a field where little is known and allowed deeper exploration of the nature of and reasons for individual attitudes to vibrations. The basis of this qualitative approach, grounded in semi-structured interviews and field observations, was that an iterative process of continuous sampling and analysis of qualitative data, such as interviews, led to the emergence of a concept or theory [30] to build an understanding of the “how and why” [29]. Subjects were purposefully sampled on the basis of their ability to inform the research question [31]. The number of participants was determined sufficient when additional interviews did not identify new theories, a point known as data saturation: this can be as few as 5–10 participants [32]. A Grounded Theory approach was used to analyse the interviews and reveal themes not previous considered [33]. Theories or concepts were identified via the practice of coding whereby transcribed material (in this study individual interviews) was labelled or annotated [34], here illustrated with two examples from this study. In the first example, almost all participants described “getting used to” the vibrations, which generated the theme of habitation and acclimatisation. In the second example, several participants expressed concern about how the vibrations affected their properties and this was then identified as a theme of worry about property damage and reduced saleability.

The study took place in the Västra Götaland and Värmland regions of Sweden among people with documented vibrations in their homes. Addresses were obtained from Trafikverket, the Swedish Transport Administration. Participants were selected from addresses within 100 m of the railway known to be exposed to vibrations. Properties with higher and lower vibration exposure levels ranging from 0.7 to 3.0 mm/s were identified with the objective of obtaining variation in the sample.

Exposure data were derived from vibration measurements executed by consulting agencies for the Swedish Transport Administration. The measurements were conducted in accordance with the Swedish guideline Dnr.S02-4235/SA60 Buller och vibrationer från spårburen linjetrafik (Noise and vibrations from intercity rail traffic) [35] and the Swedish Standard SS 460 48 61 [36]. A foundation measurement, taken from the external wall of the property adjacent to the railway, was recorded using a geophone. If the recorded foundation levels exceeded 0.6 mm/s then a follow-up measurement was taken inside the building (Swedish comfort measurement). The Swedish comfort measurement is measured in mm/s rms slow and is frequency weighted. Foundation measurements were recorded over a period of two to seven days and comfort measurements were recorded over seven days. Comfort measurements in the properties included in this study ranged from 0.7 to 3.0 mm/s.

Residents were initially contacted by letter one by one, explaining the purpose of the study and asking for their participation. Those who answered positively by sending in a written reply were contacted by telephone so that they could ask questions and make an appointment for an interview in their homes. All participants signed informed consent forms. The study was conducted in accordance with the rules of the Declaration of Helsinki of 1975 [37]. Ethical permission to investigate the way in which residents perceive and report vibrations from railways through a recorded, semi-structured interview was sought with the Swedish Central Ethical Review Board (Etikprövningsnämnderna) in Gothenburg. Ethics were granted in August 2016 (DNR 662-16).

A semi-structured interview approach was undertaken within a framework of predetermined questions. An interview guide was used, containing open questions that allowed participants to answer freely. The questions are included as an appendix. These questions were based upon findings from the Cargovibes study and factors known to generate concerns in relation to living close to wind turbines and railways [20,38]. The interviews started with general questions concerning the house, how long they had lived at their current address and their general perception of the neighbourhood. The questions were subsequently focused on the impact of the railway, first more general (e.g., “What did you think about living next to the railway when you first moved here?”, “What do you think about living next to the railway nowadays”?), and then directed towards vibrations in terms of exposure frequency (e.g., “How often do you notice vibrations inside the house?”), physical experiences (e.g., “In what way do you notice vibrations in your house?”), possible consequences (e.g., “Is your sleep disturbed?”), and possible actions (e.g., “Have you changed anything in the house due to vibration exposure?”). After analysing the first five interviews, questions regarding worry about damage to the property and the effect on the value of the property were added (e.g., “Do you think that vibrations affect the property’s value”), as was a question asking whether vibrations or noise was more problematic.

Letters were sent to 57 addresses in the Västra Götaland and Värmland regions. Fifteen replies were received giving a 26.3% response rate. Two individuals could not be reached when contacted and one declined to participate after the initial phone call. Interviews at eleven addresses with 17 individuals were arranged and conducted.

All interviews took place in the participants’ homes and were conducted in Swedish by the same researcher (first author). In seven interviews, one person in the household was present, in five interviews two people (man and woman). The interviews lasted between 25 and 60 min. The interviews were digitally recorded and transcribed on a continuous basis. 

The analyses were carried out on an ongoing basis as the interviews were transcribed in accordance with Grounded Theory. Two researchers analysed the material in parallel. Content analysis was used to capture descriptions of factual matters asked for in the interviews, that is, experience, response and effects of vibrations as phrased by the participants when they answered pre-set questions during the interview. This approach allowed the objective, systematic and repeatable identification of content inherent to the text being analysed [39]. Themes and ideas raised by participants (in this instance such as concern of property damage or worry about family members, which were talked about by several participants) were identified as emerging themes. These themes emerged in an iterative, constant comparison process in which codes were classified into themes, which in turn were adjusted by testing them in the transcribed dataset. Through this, hypothesis and conceptual frameworks for further analysis were generated. Participant recruitment, data collection and analyses continued until no new themes emerged and the identified themes were assessed as saturated, whereby emerging themes were repeated by subsequent participants and no new themes emerged. In total, 17 people in 11 households participated in this study. 

## 3. Results

### 3.1. Participants

The interviewees ranged from 41 to 82 years of age; median age 67. Of the participants, 53% were men and 47% women; 94% were married or cohabiting; 65% were pensioners and 30% in paid employment. All participants lived in a detached house with the exception of one who lived in a second floor flat. Demographics are reported in Table 1.

All interviews were conducted in participants’ homes. The interviews started with questions about the surrounding area and attitude to the railway (see interview questions in Appendix A). In general participants were positive about the area around their property. A quiet rural location was a benefit for many. 

“We like this area.”

“It’s a calm area. The only thing that’s disturbing is the railway.”

“I like it very much…it’s out of town and close to the water.”

“I like it very much. Apart from all the noise.”

“It’s a very good area…It is calm, it’s attractive and it’s central. We’re close to the post office, supermarket, doctor, pharmacy.”

“We have very good neighbours too.”

“(The thing I like most about the area) is the freedom to do what I want…we can move around freely, which we can’t do in town.”

“If you want to get out into nature you’re not far from it.”

“We are close to town but out in the countryside…with nature all around.”

“We like it very much. It’s perfect for us…It’s a little quiet and attractive and you can move about. There are no neighbours.”

“It’s the view. It’s easy to get a good view.”

When asked about their attitude to the railway participants were generally positive. Many had not given consideration to it when they first moved into the property, however they expressed a thought that the amount of traffic, in particular freight, had increased over the years and they were more disturbed by this than previously.

“We lived next to a railway before so we had nothing against it.”

“We thought there was no harm living with the railway.”

“We didn’t think about it.”

“Really it was because of it (the railway) that we moved here.”

“We didn’t think about it at all. We wanted to move to the countryside.”

“It doesn’t disturb me. It doesn’t bother me at all.”

“We didn’t think about it particularly.”

“There is more (rail) traffic, of course.”

“They take a lot more freight nowadays. And they’ve increased the speed.”

“The only thing is that they travel very fast.“

“We chose to live here. So it feels like we have accepted it.”

“Of course, it would be better without the railway, you can’t escape it, but still I don’t think it’s a big problem.” 

### 3.2. Experience of Vibrations

The experience of vibrations was described in tangible, physical terms through different senses. In contrast to noise, where one sense is stimulated, vibrations were experienced in a more multidimensional way through a number of senses. Vibrations and their effects were seen, heard and felt both in person and in the surrounding environment.

The visual impact of vibration effects was significant and perhaps the most dominant of all the senses affected. One participant described the way that vibrations were experienced as almost entirely optical and that objects could almost be seen to be vibrating. Visual effects were largely described in the context of objects in the house, as well as the house itself. Moveable objects such as porcelain and glasses were noticed to migrate over a period of time from where they had been placed in cabinets. Pictures on walls would begin to hang skewed and would need to be straightened. Several participants described seeing damage to the property itself in the form of cracks and splits in the house walls. There was a temporal dimension to the visual effects. Some were described as occurring gradually due to regular passing of trains over time, such as cracks in walls, and others were observed concurrent with the passing of a train, such as shower cubicle walls, wardrobe doors or the bed shaking.

“Porcelain in the cupboards shakes considerably.”

“Glasses in the display cabinet begin to move a bit.”

“Pictures move and need to be straightened.”

“Cracks (in the house walls) appear directly after wallpapering.”

“The display cabinet and wardrobe (upstairs)…knock against the walls.”

“The tv picture gets interference.”

The aural experience of vibrations was also described in terms of the physical environment and often related to hard, unfixed objects, such as porcelain or glasses. The words *tinkle* and *clink* were used by a number of participants to describe the sound of glasses in cabinets touching one another when trains passed. Some participants described this in almost aggressive terms with porcelain items knocking against each other and even breaking after very forceful vibrations. One participant described that in the past their record player would skip when playing a record as a train passed, disrupting the music. 

“Well, you hear it.”

“Glasses chink.”

“The glasses (…) rattle.”

“Porcelain knocks against itself.”

Participants described the feeling of vibrations in very physical terms, often when sitting or lying still, and they were commonly experienced as more pronounced higher up in the building. Some participants described feeling the bed shake at night, and even a feeling that a train was approaching because the bed began to shake long in advance of it passing. Vibrations were also felt in the entire body, not only inside the house but also when standing outside on the lawn. The way in which the feeling of vibrations in the body was described was made in comparison to different experiences but each depicted a similar sensation.

“You can feel it here (hand on chest).”

“It feels like a small earthquake.”

“Like driving over a badly gravelled road.”

“Like driving on a motorway rumble strip.”

“It’s like lying on a massage mattress.”

“The sofa moves a little (when you are sitting watching tv).”

“You can feel it inside your body. It can be a bit irritating sometimes actually.”

“The whole house shakes.”

“It’s like an earthquake’s aftershock.”

### 3.3. Response to Vibrations

#### 3.3.1. Emotional Response to Vibrations from Trains

There was an emotional response to the experience of being exposed to vibrations and their impacts. There was a feeling of being disconcerted, if vibrations were out of the ordinary or more than usual and some residents described that being able to see the trains was important in terms of not being surprised or caught off-guard. Some participants expressed a sense of irritation, in particular about the physical effects both in terms of themselves and the property.

“You begin to wonder what it is if it’s really shaky.”

“You notice every train but don’t think about it.”

“Worried about what it’s doing to the property.”

“I don’t sit and listen out for the trains.”

“You get a bit sad…it’s not so fun.”

“You get a bit sad… It’s not fun to see that…there is so much work to do in the house (because of damage).”

“I can feel a bit irritated. Worried.”

“A little unease that can lead to a little anxiety… perhaps I get a little worried (when the freight trains pass).”

#### 3.3.2. Sleep Effects Due to Vibrations from Trains

In general participants described that their sleep was not disturbed by vibrations from trains. In very exceptional cases, however, with an open window and a heavy, unbalanced train some participants did report that they awoke from sleep. On deeper consideration during the interview a small number of participants reflected that it was possible their sleep was affected by trains subconsciously. 

“I think my sleep is affected, subconsciously.”

“I think it disturbs (my sleep). Because when we’re away, and we’re sleeping elsewhere for some days, then we sleep really well.”

“I think we have worse sleep since we moved here (next to the railway)…We’ve been very tired since we moved here.”

“It’s not that I get woken by (a train) but if you are lying there awake then you react.”

“…If the window is open and a heavily-loaded, unbalanced train (passes). That can wake you up.”

### 3.4. Emerged Themes

#### 3.4.1. Habituation or Acclimatisation to Vibrations from Trains

Becoming acclimatised to the effects of vibrations was a very prominent theme, with almost all participants explaining that you get used to living next to a railway and the consequences arising from this. One participant remarked that you do not notice most of the trains, adding that this is how it is when you have lived next to a railway for a long time. This was particularly marked in comparison to guests not used to living close to a railway who notice the trains and comment about their proximity or notice that pictures are skewed where residents do not.

“You’re so used to it that you don’t think about it anymore.”

“We’re so used to it (vibrations) we hardly react.”

“We don’t wake up at night because of the trains…not now but in the beginning we did.”

“I’ve got used to it.”

“I don’t really think about it.”

“You just get so used to it. It’s just like, ok now comes another train.”

“Quite simply, we don’t care anymore.”

“Those people who are so unaccustomed to being…so close (to the railway), I think they think about it (vibrations) much more than those of us who live close do.”

“People come here and always ask, how can you live next to a railway. Well, I say, it’s fine, maybe it was annoying to begin with but, like I say, you get used to it.”

“We don’t react or think about it, or even register it (the trains).”

#### 3.4.2. Acceptance

Participants described a general sense of acceptance of the vibrations but there appeared to be a negative underlying worry, in particular about the effects on the property. However, participants did not appear to reflect on the potential health impacts of exposure to vibrations.

“I’m not worried they (the vibrations) will hurt me.”

“I don’t have any discomfort (from the vibrations).”

“I’m not troubled by the vibrations.”

“I’m not worried it’s going to hurt me.”

Participants overwhelmingly accepted that living in proximity to a railway had a certain degree of impact on lifestyle. There was also a general acknowledgement that it was their own choice to live in this way. There was a degree of acceptance of this, whether through lack of choice, resignation or pragmatism. This appears to manifest in the form of a sort of metaphorical distancing from negative consequences or experiences.

“Sometimes I think that (the cracks in the walls) are bigger, but perhaps it’s me that’s imagining it.”

“I don’t really think there has been that much to complain about.”

“I don’t dare to think about (the future).”

“there is nothing you can do (to avoid vibrations).”

“It’s the way it is when you live close to the train.”

Noise was overwhelmingly considered more disturbing than vibrations, however there was a strong sense that measures could be taken and behaviour changed to avoid noise whereas a sense of helplessness and lack of empowerment to avoid vibrations emerged. 

“That’s just how it was.”

“It’s something you just can’t do anything about.”

“We can’t do anything about this noise or the vibrations.”

“To get them to stop running the trains, I don’t think that can happen.”

#### 3.4.3. Worry about Property Damage and Reduced Saleability

Worry about factors other than exposure to vibrations was a dominant theme that emerged during the course of the interviews.

A recurring and prominent theme was worry about the property. This was both in terms of damage resulting from vibrations and proximity to the railway track and, in particular, to the negative implications of this and not being able to sell the property. This was a particularly conspicuous theme among older participants, who expressed a feeling of wanting freedom to move in the near future as they got older, but of feeling that they would be unable to do so, as they would not be able to sell the property or would be burdened with outstanding debt. This was described as being very nerve-wracking and unpleasant. The word *prison* was also used by three separate participants. 

“The house doesn’t benefit from the shaking and vibrating.”

“Perhaps it’s not good for the house itself.”

“Sometimes you wonder what all this shaking is doing (to the house).”

“The house isn’t benefitting from such vibrations.”

“Personally, I don’t have a problem with trains. The only problem I have is with the property. That’s the bit I see as a drawback.”

“Worried about…what it’s doing to the property.”

”I think if we had a house viewing today and a freight train came passed then I think that 75 percent of the viewers would turn on their heel and go.”

“Some of the windows can’t be opened and I think it’s because of the shaking. Same with the outer door now that I think about it.”

“It’s mostly the house that’s affected by vibrations, not so much us.”

#### 3.4.4. Worry about Well-Being of Family Members

Many of the participants expressed concerns about family members and visitors that came to their homes rather than concern for themselves. The main thrust of this worry centred around safety and well-being. This was often expressed in the context of children that were staying and appeared to be associated with a sense of sadness or guilt.

“The grandkids don’t really dare to sleep with open windows.”

“My niece was almost hysterical (when she felt the vibrations).”

“My niece (thought it was) an earthquake. She was totally beside herself.”

“They (the vibrations) are unpleasant for them (the grandchildren).”

“When the grandkids stay over…they get scared… It was ok to begin with but now they listen out for the trains.”

This worry about well-being of others had social consequences and impinged on a freedom to be able to behave freely. It generated a strong emotional response in several participants, including a sense of shame and guilt.

“I think it’s embarrassing.”

“I’m ashamed.”

“It’s not nice that the grandkids get scared, you want them here anyway, to sleep over.”

#### 3.4.5. General Safety of Living Close to the Line Related to Fires or Derailing

The safety of the railway emerged as a significant theme and went beyond the scope of vibrations to the possibility of more catastrophic repercussions, including train derailment and fire. These thoughts concerned a number of residents. The railway was also viewed as an infringement on behaviour. One participant said they had not wanted to dig a deep well for water for fear that vibrations would cause it to be damaged and let in soil. They took a more expensive, less-preferred option and decided to connect to local authority water supply instead. The railway was also seen as a physical barrier by one participant who expressed that physically getting passed the railway was “not that cool”. 

“I was most worried that they (the grandchildren) would get out (out of the garden)…that was a bit unpleasant, thinking they could fall over (onto the track).”

“There are other worrying thoughts that are much worse in fact. What would happen if an X2000 (fast train) derails at 200 kph? It would ram the whole house, every house here.”

“Yes, I am worried that is, I think that sooner or later a train is going to derail.”

“There is a risk living together with the railway. That is clear.”

## 4. Discussion

Environmental stressors, such as noise and air pollution, are typically chronic in nature, physically perceptible and unavoidable. They are often viewed negatively by those who experience them [40] and give rise to negative emotions, including higher levels of annoyance [25,41,42].

This study examined the perception of individuals living close to a railway to vibrations from rail traffic and experiences connected to them. As with other types of environmental stressors, participants described being exposed chronically to these vibrations, every day for the duration of their time living in their property. Almost all were physically aware of vibrations in the property at some time during the day, whether inside the house or in the garden, and some felt them in their body. Several participants expressed worry about the effects on belongings or the house associated with the vibrations, in spite of the fact that the measured levels appear to lie within accepted levels in the building standard [43].

That this stressor is experienced inside participants’ properties is important. The concept of the home as a secure sanctuary and its role in restoration is significant here, particularly in the context of the WHO holistic health definition [16]. Stress, restoration and coping can be viewed as a triad of inter-related concepts [44]. Stress is a complex notion perhaps here best viewed as the imbalance between external and personal demands placed on an individual and the availability of biopsychosocial resources to meet them [44,45]. Managing stress causes a depletion in physical, social and psychological resources and restoration is an opportunity to recover them [44]. Sleep is a basic and fundamental form of restoration, allowing renewal of physical and mental capacity. During waking hours restoration can be achieved in two ways: creating distance from stressors and demands, and gaining respite in a pleasing environment [44].

It is suggested that a lack of ability for restoration within the home and inability for respite from an environmental stressor is negative for health [38]. There are two potential sources of disruption to the home’s role in restoration in the context of vibrations from rail. Experimental studies have shown that sleep is disrupted by the experience of being exposed to vibrations [46]. If vibrations can be felt at regular intervals when sitting or lying still this may be disruptive in moments of relaxation and may indicate a reduced possibility of psychophysiological restoration.

Furthermore, studies examining annoyance in relation to noise show that that access to quiet spaces is associated with reduced annoyance [47], which suggests that space away from the environmental stressor has a protective role to play for negative emotion. The lack of available space in which to avoid vibrations in the home may therefore inhibit the possibility for restoration and maintain, or perhaps exacerbate, a feeling of annoyance. In addition, the duration of time over which individuals are exposed is often more significant in terms of perceived annoyance than the noise level itself [47]. If this pattern is replicated in the context of vibration exposure both the chronic nature of exposure and the inability to avoid vibrations may contribute to heightened negative emotions.

This potential reduced ability for restoration and increased negative emotion associated with chronic exposure to environmental stressors, here vibrations, in individuals’ living environments may negatively impact health over the longer term. Moreover, the underlying worry about general well-being and safety which emerged as a prominent theme in this study, is also likely to negatively impact health. 

Although difficult to evidence causation, it has been suggested that perseverative cognition (continuous thinking about past, current or future negative events), manifesting as worry and rumination in response to actual or anticipated stress, is associated with increased cardiovascular and endocrine pathology [48]. This may be mediated through disruption to the HPA axis. The way in which we as individuals respond to and manage stress is mediated by our cognitive coping strategies which affects our physiological reaction. Restoration is important as its absence can compromise our ability to fully perform this coping strategy [4].

Although worry emerged as a theme in the study, participants did not reflect on the impact on their individual health and well-being. This may represent a coping mechanism to manage exposure in the form of avoidance. Trait-orientated coping mechanisms, described by Lazarus, focus on individual coping methods and resources and encompass two central constructs to explain an individual’s coping strategy to stress: vigilance, or positioning oneself to face the stressor (a more active role); and cognitive avoidance, or aversion of the source of stress (a more passive role) [49].

Chronic exposure to environmental stressors, such as noise, has been associated with a sense of helplessness and has also been linked to feelings of depression [50]. Exposure to minor but accumulated daily irritations may in fact be more stressful than big life events, such as bereavement and divorce [4], particularly when embedded in a lack of empowerment to affect change where no end point is perceived. Helplessness to affect changes to vibration exposure was a prominent theme in this study, and was specifically related to vibrations. This contrasted to noise, where there was a feeling of being able to put measures in place to reduce exposure. Controllability of a stressor is a key factor in the way in which we evaluate it [4]. Inability to control, or escape, stressors and shape our environment, is associated with a greater perception of stress. In order to manage this, individuals may then have to draw more heavily from their personal resources in order to cope.

Individuals might ordinarily adopt coping mechanisms that involve a more active role, implementing changes to remove the stressor—often a successful strategy as seen with the installation of, for example, triple glazing to mitigate noise pollution. In the case of exposure to rail vibrations there appears to be a lack of ability to implement changes to reduce vibrations [51]. Asked if they do something to avoid vibrations, the response in the study group was consistently as one participant said, “No, we can’t do anything about them”. Instead individuals appear to adopt other strategies, such as avoidance and acceptance of their circumstance. This sense of powerlessness to limit or remove exposure and therefore inability to mitigate worry about well-being and damage to property could, therefore, affect health negatively through perseverative cognition.

It is suggested that exposure to intractable environmental stressors, such as noise, require the use of high levels of an individual’s personal resource in order to cope [52]. If unable to draw on sufficient resources this may undermine the sense of security with the home environment. The feelings of helplessness expressed by many participants were linked to acceptance of the living environment, as well as a sense of frustration. This may arise from a need to maintain a psychological distance from a perceived disturbance and may also represent a coping mechanism in the form of acceptance that limited or no measures can be taken to counteract exposure to vibrations.

Although evidence is mixed, perceived stress has been associated with elevated cortisol levels [53]. Perception of stress is based on previous experience and expectations of what will occur. Feedback from this stress response is affected and modified by coping mechanisms and strategies. If coping strategies are not successful this may result in individuals feeling additional stress. This may in turn have negative repercussions for the HPA axis through a directly-acting pathway whereby the body’s “flight or fight” response is activated resulting in upregulation of the sympathetic nervous system and, for example, an increase in heart rate or blood pressure. This puts physiological strain on individuals, which may predispose them to cardiovascular morbidity.

Furthermore, there is a connection between the way in which people describe what happens in their homes and their consequent emotional response. This may represent an indirect path to disrupt the HPA axis that in the long-term could cause ill-health. Whether acting independently or symbiotically these direct and indirect pathways could give rise to longer-term health problems. Further research to identify possible health risks, such as cardiovascular disease, arising secondary to exposure to vibrations would help to elucidate these potential underlying pathways.

Vibrations are experienced in a multi-dimensional way through three different senses: sight, sound and somatosensation. Research shows that visual perception negatively influences an individual’s annoyance level in response to noise from wind turbines [22,54]. It is possible that a similar pattern is seen with annoyance or coping levels in response to vibrations, whereby seeing, hearing and feeling the effects of vibrations, such as rattling and movement, may increase annoyance or worry. Furthermore, as the experience of vibrations is multi-modal this effect may be amplified and lead to a decreased ability to cope with them, known as a potentiating effect [50]. This suggests that vibrations cannot be assessed in the same way as noise, as different modifiers come into the picture. Further research is needed to understand the effects of exposure to multiple stressors.

One of the strengths of this study was that it allowed in-depth exploration of the description of and perception to vibrations in participants’ home environments. The use of both content analysis and a Grounded Theory approach allowed both factual descriptions and emerging themes to be captured. Consistency in analysis was ensured by making comparison of themes in interviews one and five, showing agreement between 19 themes and a difference with two themes where consensus was reached after subsequent discussion. One of the possible limitations is that quotations were translated from Swedish to English by the first author, and while objectivity was maintained, this should be noted.

## 5. Conclusions

Vibrations from rail transport are experienced by those living in proximity to the source through a number of senses. Residents in this study had lived in the property in the long-term, for up to 50 years, and described being exposed to vibrations regularly, several times over the course of a day, every day. The chronic nature of exposure to this environmental stressor coupled with the multimodal experience may increase a sense of being exposed. It may even be that vibrations are associated with a perception of being more “all-encompassing” and difficult to avoid. Indeed, this inability to avoid or mitigate vibrations was expressed by almost all participants and may have significant negative repercussions for the individual ability for restoration. Respite from stressors is an important component of restoration, a vital process that helps restore resources needed to manage the demands of stress. The potential lack of possibility for restoration in the home has negative implications for maintaining health and well-being. Strategies to cope with exposure to vibrations are important and those that seek to remove the stressor appear not to be possible in the case of vibrations from rail. Other coping strategies, including avoidance, may be adopted. However, ongoing worry about current or future consequences of vibration exposure may have longer-term negative health effects, as a result of disruption to the body’s normal homeostatic stress response.

## Figures and Tables

**Table 1:** Participant demographics.

**Sex**	Women n (%)	8 (47)
**Age Range (Median)**		41–82 (67)
**Marital Status**	Married/cohabiting n (%)	16 (94)
	Widow/widower n (%)	1 (6)
**Occupation**	Employed full time n (%)	3 (18)
	Employed part time n (%)	2 (12)
	Long-term sick n (%)	1 (6)
	Pensioner n (%)	11 (65)
**Years Lived at Property Range (Median)**		5–50 (28.5)

## Appendix A

**First questions:**

How long have you lived here?

Do you like living in this area? Why?

What did you think about living next to the railway when you first moved here?

What do you think about living next to the railway nowadays?

How do think it will be in the future?

Do you hear trains inside the house?

How often?

What time of day?

Do you hear them at any particular time?

How does it affect you?

Do you notice vibrations in the house?

How often?

What time of day?

Do you notice them at any particular time?

In what way do you notice the vibrations?

Does it affect the house? In what way?

Do the vibrations affect you? In what way? Is your sleep disturbed?

To those who are disturbed:

What do you do when the noise disturbs you? Have you changed anything in the house? Have you had contact with the authorities? Have you changed your behaviour or lifestyle?

What do you do to avoid vibrations? Have you changed anything in the house? Have you had contact with the authorities? Have you changed your behaviour or lifestyle?

To those who are not disturbed:

You don’t experience any problems in your house but others do. What do you think is the reason that there are problems in some houses but not yours?

**Questions added after analysis of the first five interviews:**

How do you feel about the thought that the house might get damaged?

Do you think that vibrations affect the property’s value?

Can you feel the vibrations in your body?

Is there anything that worries you in relation to the vibrations?

How do you feel when you think about… (the thing that worries you)?

What do you think is the bigger problem—noise or vibrations?

## References

[1] European Commission (2011). Roadmap to a Single European Transport Area—Towards a Competitive and Resource Efficient Transport System.

[2] Shift2Rail Horizon 2020 European Union Funding for Research and Innovation. https://shift2rail.org/about-shift2rail/.

[3] Kuller R., Gärling T., Evans G.W. (1991). Environmental Assessment from a Neuropsychological Perspective. Environment, Cognition and Action: An Integrated Approach.

[4] Cassidy T., Cochrane R. (1997). Environmental Psychology: Behaviour and Experience in Context.

[5] Parati G., Antonicelli R., Guazzarotti F., Paciaroni E., Mancia G. (2001). Cardiovascular effects of an earthquake: Direct evidence by ambulatory blood pressure monitoring. Hypertension.

[6] Lin L.-Y., Wu C.-C., Liu Y.-B., Ho Y.-L., Liau C.-S., Lee Y.-T. (2001). Derangement of heart rate variability during a catastrophic earthquake: A possible mechanism for increased heart attacks. Pacing Clin. Electrophysiol..

[7] Bland S.H., Farinaro E., Krogh V., Jossa F., Scottoni A., Trevisan M. (2000). Long term relations between earthquake experiences and coronary heart disease risk factors. Am. J. Epidemiol..

[8] Ursin H., Eriksen H.R. (2004). The cognitive activation theory of stress. Psychoneuroendocrinology.

[9] Ursin H., Eriksen H.R. (2010). Cognitive activation theory of stress (CATS). Neurosci. Biobehav. Rev..

[10] Oxford Dictionaries. http://www.oxfordreference.com/search?q=allostasis&searchBtn=Search&isQuickSearch=true..

[11] McEwen B.S. (2004). Protection and damage from acute and chronic stress: Allostasis and allostatic overload and relevance to the pathophysiology of psychiatric disorders. Ann. N. Y. Acad. Sci..

[12] Munzel T., Gori T., Babisch W., Basner M. (2014). Cardiovascular effects of environmental noise exposure. Eur. Heart J..

[13] World Health Organization (2011). Burden of Disease from Environmental Noise, Quantification of Healthy Life Years Lost in Europe.

[14] Guski R., Felscher-Suhr U., Schuemer R. (1999). The concept of noise annoyance: How international experts see it. J. Sound Vib..

[15] Lindvall T., Radford E.P. (1973). Measurement of annoyance due to exposure to environmental factors. Environ. Res..

[16] Constitution of WHO: Principles 1946. http://www.who.int/about/mission/en/.

[17] Beutel M.E., Junger C., Klein E.M., Wild P., Lackner K., Blettner M. (2016). Noise annoyance is associated with depression and anxiety in the general population—The contribution of aircraft noise. PLoS ONE.

[18] Waddington D., Woodcock J., Smith M.G., Janssen S., Persson Waye K. (2015). Cargovibes: Human response to vibration due to freight rail traffic. Int. J. Rail Transp..

[19] Licitra G., Fredianelli L., Petri D., Vigotti M.A. (2016). Annoyance evaluation due to overall railway noise and vibration in Pisa urban areas. Sci. Total Environ..

[20] Peris E., Woodcock J., Sica G., Sharp C., Moorhouse A.T., Waddington D.C. (2014). Effect of situational, attitudinal and demographic factors on railway vibration annoyance in residential areas. J. Acoust. Soc. Am..

[21] Persson Waye K., Öhrström E. (2002). Psycho-acoustic characters of relevance for annoyance of wind turbine noise. J. Sound Vib..

[22] Zhang B., Shi L., Di G. (2003). The influence of the visibility of the source on the subjective annoyance due to its noise. Appl. Acoust..

[23] Aletta F., Masullo M., Maffei L., Kang J. (2016). The effect of vision on the perception of the noise produced by a chiller in a common living environment. Noise Control Eng. J..

[24] Maffei L., Masullo M., Aletta F., Di Gabriele M. (2013). The influence of visual characteristics of barriers on railway noise perception. Sci. Total Environ..

[25] Pedersen E., Persson Waye K. (2007). Wind turbine noise, annoyance and self-reported health and well-being in different living environments. Occup. Environ. Med..

[26] Pedersen E., Larsman P. (2008). The impact of visual factors on noise annoyance among people living in the vicinity of wind turbines. J. Environ. Psychol..

[27] Basner M., Brink M., Bristow A., de Kluizenaar Y., Finegold L., Hong J. (2015). ICBEN review of research on the biological effects of noise 2011–2014. Noise Health.

[28] Quehl J., Muller U., Mendolia F. (2017). Short-term annoyance from nocturnal aircraft noise exposure: Results of the NORAH and STRAIN sleep studies. Int. Arch. Occup. Environ. Health.

[29] Sullivan G.M., Sargeant J. (2011). Qualities of qualitative research: Part I. J. Grad. Med. Educ..

[30] Pidgeon N., Richardson J.T.E. (1996). Grounded Theory: Theoretical background. Handbook of Qualitative Research Methods.

[31] Sargeant J. (2012). Qualitative research part II: Participants, analysis, and quality assurance. J. Grad. Med. Educ..

[32] Fusch P.I., Ness L.R. (2015). Are we there yet? Data saturation in qualitative research. Qual. Rep..

[33] Glaser B., Strauss A. (1967). The Discovery of Grounded Theory: Strategies for Qualitative Research.

[34] Pidgeon N., Henwood K., Richardson J.T.E. (1996). Grounded Theory: Practical Implementation. Handbook of Qualitative Research Methods.

[35] Buller Och Vibrationer Från Spårburen Linjetrafik—Riklinjer Och Tillämpning. https://www.trafikverket.se/contentassets/91a9a5fa41d4449ea2c06512dfc63605/buller_vibr_riktlinj_omarb_version_20060201.pdf.

[36] Vibration Och Stöt—Riktvärden Och Mätmetod för Vibrationer I Byggnader Orsakade av Pålning Spontning, Schaktning Och Packning. https://www.sis.se/produkter/metrologi-och-matning-fysikaliska-fenomen/vibrationer-och-stot-vibrationsmatning/ss25211/.

[37] Declaration of Helsinki: Medical Research Involving Human Subjects 2013. https://www.wma.net/what-we-do/medical-ethics/declaration-of-helsinki/.

[38] Pedersen E., Waye K.P. (2008). Wind turbines—Low level noise sources interfering with restoration?. Environ. Res. Lett..

[39] Krippendorff K. (2012). Content Analysis: An Introduction to Its Methodology.

[40] Campbell J.M. (1983). Ambient stressors. Environ. Behav..

[41] Van Kamp I. (2016). A Systematic Review of Evidence of the Effect of Transport Noise Interventions on Human Health.

[42] Melo M., Santos J.M., Frere S., Reisen V., Reis N.C., Leite M.F.S. (2015). Annoyance caused by air pollution: A comparative study of two industrialized regions. World Acad. Sci. Eng. Technol. Int. J. Environ. Ecol. Eng..

[43] ISO (2010). Standards Policy and Strategy Committee. Mechanical Vibration and Shock—Vibration of Fixed Structures—Guidelines for the Measurement of Vibrations and Evaluation of Effects on Structures.

[44] Hartig T., Johansson G., Kylin C. (2003). Residence in the social ecology of stress and restoration. J. Soc. Issues.

[45] Institute for Stress Medicine The ISM Definition of Stress: Institute for Stress Medicine. http://www.vgregion.se/ov/ISM/stress--rad-och-behandling/vad-ar-stress/definition-pa-stress/.

[46] Smith M.G., Croy I., Hammar O., Persson Waye K. (2016). Vibration from freight trains fragments sleep: A polysomnographic study. Sci. Rep..

[47] Van Kamp I., Davies H. (2008). Environmental Noise and Mental Health: Five Year Review and Future Directions. 9th International Congress on Noise as a Public Health Problem (ICBEN).

[48] Brosschot J.F., Gerin W., Thayer J.F. (2006). The perseverative cognition hypothesis: A review of worry, prolonged stress-related physiological activation and health. J. Psychosom. Res..

[49] Krohne H.W., Smelser N.J., Baltes P.B. (2001). Stress and Coping Theories. International Encyclopedia of the Social and Behavioral Sciences.

[50] Evans G.W., Stecker R. (2004). Motivational consequences of environmental stress. J. Environ. Psychol..

[51] De Freitas R.T., Kaewunruen S. (2016). Life cycle cost evaluation of noise and vibration control methods at urban railway turnouts. Environments.

[52] Riedel N., Köckler H., Scheiner J., Berger K. (2013). Objective exposure to road traffic noise, noise annoyance and self-rated poor health—Framing the relationship between noise and health as a matter of multiple stressors and resources in urban neighbourhoods. J. Environ. Plann. Manag..

[53] Takahashi T., Ikeda K., Ishikawa M., Kitamura N., Tsukasaki T., Nakama D. (2005). Anxiety, reactivity, and social stress-induced cortisol elevation in humans. Neuroendocrinol. Lett..

[54] Pedersen E., Waye K.P. (2004). Perception and annoyance due to wind turbine noise—A dose-response relationship. J. Acoust. Soc. Am..

